# The Sunlight-Vitamin D Connection: Implications for Patient Outcomes in the Surgical Intensive Care Unit

**DOI:** 10.7759/cureus.46819

**Published:** 2023-10-10

**Authors:** Tapesh D Nagaria, Raju K Shinde, Samarth Shukla, Sourya Acharya, Neema Acharya, Sangita D Jogdand

**Affiliations:** 1 General Surgery, Jawaharlal Nehru Medical College, Datta Meghe Institute of Higher Education and Research, Wardha, IND; 2 Pathology, Jawaharlal Nehru Medical College, Datta Meghe Institute of Higher Education and Research, Wardha, IND; 3 Medicine, Jawaharlal Nehru Medical College, Datta Meghe Institute of Higher Education and Research, Wardha, IND; 4 Obstetrics and Gynaecology, Jawaharlal Nehru Medical College, Datta Meghe Institute of Higher Education and Research, Wardha, IND; 5 Pharmacology and Therapeutics, Jawaharlal Nehru Medical College, Datta Meghe Institute of Higher Education and Research, Wardha, IND

**Keywords:** critical care, supplementation, immunomodulation, patient outcomes, surgical intensive care unit (sicu), vitamin d

## Abstract

This review delves into the intricate relationship between Vitamin D and patient outcomes in the Surgical Intensive Care Unit (SICU). Vitamin D, known for its multifaceted roles in immune modulation, inflammation regulation, and maintenance of calcium homeostasis, emerges as a pivotal factor in the care of critically ill patients. Our exploration reveals a high prevalence of Vitamin D deficiency in the SICU, primarily attributable to limited sunlight exposure, comorbidities, and medication use. Importantly, Vitamin D status impacts infection rates, mortality, and length of stay in the SICU, making it a clinically relevant consideration. Mechanistic insights into the immunomodulatory and anti-inflammatory effects of Vitamin D shed light on its potential benefits in critical care. However, challenges, including accurate assessment, individualised supplementation, and ethical considerations regarding sunlight exposure, are evident. The prospect of personalised Vitamin D supplementation strategies offers promise for optimising patient care. In conclusion, the Sunlight-Vitamin D Connection holds significant potential to improve outcomes in the SICU, emphasising the importance of further research and tailored approaches for the well-being of critically ill individuals.

## Introduction and background

Vitamin D, often called the "sunshine vitamin," is a fat-soluble secosteroid that plays a crucial role in maintaining human health. It is unique among vitamins because it can be synthesised in the skin in response to sunlight exposure, making it an essential nutrient and a hormone. Over the years, research has unveiled many functions attributed to Vitamin D within the body. While its classical role is well-known for regulating calcium and phosphorus homeostasis to support bone health, emerging evidence suggests that Vitamin D exerts a much broader influence on various physiological processes [1,2].

Beyond its classical role, Vitamin D has been implicated in numerous aspects of health. It is a potent immunomodulator, influencing the innate and adaptive immune responses, which have sparked interest in its potential role in various disease states. Epidemiological studies have linked Vitamin D deficiency to various health conditions, including osteoporosis, autoimmune diseases, cardiovascular diseases, and cancer. Moreover, Vitamin D is crucial for the functioning of the nervous system and has been associated with mental health disorders. This multifaceted significance has driven substantial research into understanding how Vitamin D impacts health and disease [3,4].

Vitamin D can be obtained through various sources, but its primary natural source is sunlight. When ultraviolet B (UVB) radiation from the sun penetrates the skin, it triggers a series of enzymatic reactions that lead to the synthesis of Vitamin D in the body. This process highlights the crucial role of sunlight exposure in maintaining adequate Vitamin D levels. However, it's important to note that dietary sources and supplements contribute significantly to Vitamin D intake, particularly in regions with limited sunlight exposure. These dietary sources include fatty fish (e.g., salmon, mackerel), fortified foods (e.g., milk, cereal), and supplements [5].

The Surgical Intensive Care Unit (SICU) is a specialised medical setting where critically ill patients receive intensive care following surgical procedures. The connection between Vitamin D status and patient outcomes in this context has become an area of growing interest among researchers and healthcare professionals. Given the diverse physiological functions of Vitamin D and its role in immune regulation, it is essential to investigate its potential impact on SICU patient outcomes [6].

This review aims to comprehensively examine the relationship between sunlight exposure, Vitamin D status, and the outcomes of patients in the SICU. By delving into the existing literature, we will explore how Vitamin D deficiency may influence infection rates, mortality, length of stay, and other clinical parameters in critically ill surgical patients. Furthermore, we will discuss the mechanisms through which Vitamin D could exert its effects in the SICU setting and highlight the clinical implications for patient care. Understanding the Sunlight-Vitamin D Connection in the SICU may offer valuable insights into optimising patient management and improving outcomes in this critical care environment.

## Review

The role of vitamin D in human health

Functions of Vitamin D in the Body

Vitamin D is a versatile molecule that influences the human body. Its functions extend beyond the traditional role of regulating calcium and phosphorus metabolism. Some essential functions include:

Calcium homeostasis: Vitamin D plays a central role in maintaining calcium homeostasis in the body. Calcium is a crucial mineral necessary for various physiological processes. Vitamin D enhances the absorption of dietary calcium in the small intestine, ensuring that an adequate amount of calcium is available in the bloodstream. This function is essential for maintaining proper calcium levels in the blood, which, in turn, is vital for bone health, muscle contraction, and nerve signalling [7].

Bone health: Vitamin D is often synonymous with bone health. It is indispensable for bone mineralisation, where it aids in incorporating calcium and phosphorus into the bone matrix. This process is pivotal in maintaining bone density and strength. Without sufficient Vitamin D, calcium absorption is impaired, weakening bones an increased risk of fractures, and conditions like osteoporosis [8].

Immune system modulation: Vitamin D acts as a potent immunomodulator, influencing the immune system's function in various ways. It plays a role in both innate and adaptive immunity. In the innate immune response, Vitamin D enhances the production of antimicrobial peptides like cathelicidins and defensins, which are essential for defence against infections. The adaptive immune response regulates the proliferation and differentiation of T and B cells, two critical immune system components. By modulating the immune response, Vitamin D can help the body defend against pathogens and may be involved in autoimmune diseases where the immune system mistakenly attacks the body's tissues [9].

Cell growth and differentiation: Vitamin D also regulates cell growth and differentiation. It has been identified as a critical player in differentiating various cell types in different tissues, including skin, prostate, breast, and colon. Proper cell growth and differentiation regulation is crucial for maintaining tissue health and preventing abnormal cell proliferation, often associated with cancer [10].

Cardiovascular health: Emerging research suggests Vitamin D may affect cardiovascular health. While the exact mechanisms are still under investigation, Vitamin D is thought to influence blood pressure regulation and endothelial function. Some studies have associated Vitamin D deficiency with an increased risk of hypertension, heart disease, and stroke. However, more research is needed to establish the precise relationship between Vitamin D and cardiovascular health [11].

Mental health: Vitamin D receptors are present in the brain, indicating a potential role in mental health. Vitamin D deficiency has been linked to mood disorders such as depression and seasonal affective disorder (SAD). The exact mechanisms behind these associations are not fully understood but may involve the influence of Vitamin D on neurotransmitters and brain function [12].

Sources of Vitamin D (Diet, Supplements, Sunlight)

Dietary sources: Dietary sources of Vitamin D are somewhat limited, and natural food options containing significant amounts of Vitamin D are relatively few. Fatty fish, such as salmon, tuna, mackerel, and sardines, are among the diet's richest sources of Vitamin D. Cod liver oil is another notable source, providing a concentrated dose of Vitamin D. In addition to these natural sources, many food products are fortified with Vitamin D to help people meet their dietary requirements. Common examples include milk, orange juice, breakfast cereals, and yoghurt, often enriched with added Vitamin D to enhance their nutritional content. These fortified foods are precious for individuals with limited access to natural sources of Vitamin D, such as those living in regions with limited sunlight exposure [13].

Supplements: Vitamin D supplements are widely used to address deficiencies and maintain optimal Vitamin D levels in individuals who may have difficulty obtaining sufficient Vitamin D from dietary sources or sunlight alone. There are two main types of Vitamin D supplements: Vitamin D2 (ergocalciferol) and Vitamin D3 (cholecalciferol). Both forms can effectively raise blood levels of Vitamin D when taken as directed. Vitamin D3, in particular, is often preferred because it is the same form produced naturally in the skin in response to sunlight exposure. These supplements are available over the counter and are prescribed by healthcare providers based on individual needs and Vitamin D status. They are essential in managing and preventing Vitamin D deficiencies, especially in individuals with limited exposure to sunlight or specific medical conditions [14].

Sunlight exposure: Sunlight exposure is a primary natural source of Vitamin D and plays a critical role in maintaining adequate Vitamin D levels. A chemical reaction occurs when the skin is exposed to ultraviolet B (UVB) radiation from sunlight. Specifically, cholesterol in the skin is converted into a precursor molecule known as 7-dehydrocholesterol. This precursor is then further transformed into Vitamin D, specifically Vitamin D3 (cholecalciferol), the biologically active form of Vitamin D. The efficiency of this process depends on various factors, including geographic location, time of day, season, and individual skin type. For instance, individuals in regions closer to the equator typically receive more intense sunlight throughout the year, allowing for more efficient Vitamin D synthesis. However, during the winter months or in regions with limited sunlight, the skin's ability to produce Vitamin D may be significantly reduced. Additionally, people with darker skin may require more extended sun exposure to produce the same amount of Vitamin D as those with lighter skin due to the natural sun-protective effects of melanin. It's essential to balance obtaining Vitamin D from sunlight exposure and protecting the skin from the harmful effects of excessive UV radiation, such as sunburn and skin cancer. Therefore, recommendations for sunlight exposure to maintain Vitamin D levels should be based on individual factors and expert guidance [2].

Metabolism of Vitamin D in the Skin

The synthesis of Vitamin D in the skin is a complex and highly regulated process that begins with the interaction of ultraviolet B (UVB) radiation with a precursor molecule called 7-dehydrocholesterol. This intricate photochemical reaction occurs in the epidermal layer of the skin. It sets in motion a series of transformations that ultimately yield the biologically active form of Vitamin D, calcitriol (1,25-dihydroxycholecalciferol).

UVB radiation exposure: The initial step in Vitamin D synthesis occurs when the skin is exposed to UVB radiation, which ranges in wavelength from approximately 290 to 320 nanometers (nm). UVB radiation is most abundant in sunlight and is responsible for triggering the synthesis of Vitamin D. When UVB rays penetrate the skin, they interact with 7-dehydrocholesterol, a naturally occurring compound found in the epidermal layer of the skin [5].

Formation of cholecalciferol (Vitamin D3): The interaction between UVB radiation and 7-dehydrocholesterol results in a photochemical reaction that transforms 7-dehydrocholesterol into cholecalciferol, commonly called Vitamin D3. This newly formed cholecalciferol is still inactive and requires further processing to become biologically active [2].

Transport to the liver and kidneys: Once cholecalciferol (Vitamin D3) is generated in the skin, it is transported through the bloodstream to the liver. In the liver, an enzyme called 25-hydroxylase converts cholecalciferol into 25-hydroxycholecalciferol, also known as calcidiol. While calcidiol is a relatively inactive form of Vitamin D, it is an intermediate product in the conversion process [15].

Final conversion in the kidneys: The final and crucial step in activating Vitamin D occurs in the kidneys. Here, another enzyme called 1-alpha-hydroxylase converts calcidiol into its biologically active form, calcitriol (1,25-dihydroxycholecalciferol). Calcitriol is the hormonally active form of Vitamin D and is responsible for carrying out the various functions of Vitamin D throughout the body [16].

Importance of Vitamin D for the Immune System

Innate immunity: Vitamin D is critical in enhancing innate immunity, the body's first defence against pathogens. It achieves this by promoting the activity of antimicrobial peptides such as cathelicidins and defensins. These antimicrobial molecules are produced by various cells, including immune cells and epithelial cells. Cathelicidins and defensins have potent antimicrobial properties, allowing them to directly combat invading pathogens like bacteria, viruses, and fungi. By boosting the production of these antimicrobial peptides, Vitamin D enhances the body's ability to fend off infections, particularly in mucosal tissues like the respiratory and gastrointestinal tracts [17].

Adaptive immunity: Vitamin D also regulates adaptive immunity, the branch of the immune system responsible for mounting particular responses to pathogens. This modulation occurs by regulating T and B cells, critical players in adaptive immune responses [18].

T cells: Vitamin D influences the differentiation and function of T cells, including both helper T cells (Th) and cytotoxic T cells (Tc). These cells are essential for orchestrating and executing immune responses. Vitamin D promotes the development of regulatory T cells (Tregs), which are crucial in suppressing excessive immune responses and preventing autoimmunity [3].

B cells: Vitamin D can also influence the function of B cells, which are responsible for producing antibodies and mediating humoral immunity. It may regulate antibody production and class switching in B cells, contributing to balanced immune responses [19].

Anti-Inflammatory Effects: Excessive inflammation is implicated in the pathogenesis of various chronic diseases, including autoimmune disorders and cardiovascular conditions. Vitamin D has anti-inflammatory properties that help modulate the immune response and reduce the risk of chronic inflammation. It can inhibit the production of pro-inflammatory cytokines, such as interleukin-6 (IL-6) and tumour necrosis factor-alpha (TNF-α). By mitigating inflammation, Vitamin D contributes to maintaining immune homeostasis and preventing immune-related disorders [20].

Sunlight exposure and vitamin D synthesis

How UVB Radiation from Sunlight Leads to Vitamin D Production in the Skin

The synthesis of Vitamin D in the skin is a fascinating and highly regulated process initiated by the interaction of ultraviolet B (UVB) radiation from sunlight with a specific precursor molecule, 7-dehydrocholesterol, which is naturally present in the skin. This process can be summarised in the following steps:

UVB radiation exposure: The first step in synthesising Vitamin D in the skin is exposure to UVB radiation. UVB radiation encompasses wavelengths between 290 and 320 nanometers and is a component of natural sunlight. When the skin is exposed to sunlight, particularly UVB rays, they penetrate the epidermal layer, the outermost layer of the skin [13].

7-dehydrocholesterol conversion: Upon exposure to UVB radiation, 7-dehydrocholesterol, a molecule that is abundant in the epidermal layer of the skin, undergoes a photochemical reaction. This reaction is induced by the energy from the UVB rays and results in the conversion of 7-dehydrocholesterol into a precursor molecule known as previtamin D3 or cholecalciferol. The molecule has undergone a structural transformation at this stage, but it is not yet in its biologically active form [5].

Thermal isomerization: The previtamin D3, or cholecalciferol, formed in the previous step is relatively unstable. It undergoes a thermal isomerisation process to become the biologically active form of Vitamin D, known as Vitamin D3 (cholecalciferol). This thermal isomerisation occurs spontaneously in response to body heat and involves rearranging the molecule's structure. As a result of this transformation, cholecalciferol becomes Vitamin D3, the form of Vitamin D that the body can utilise for various physiological functions [21].

Factors Influencing Sunlight-Mediated Vitamin D Synthesis

Latitude: The distance north or south of the equator significantly affects the intensity of sunlight and UVB radiation levels. Regions closer to the equator receive more direct and intense sunlight throughout the year. Consequently, people living in equatorial regions generally have more opportunities for Vitamin D synthesis. In contrast, regions at higher latitudes, farther from the equator, experience reduced UVB exposure, especially during the winter months. This reduced exposure can limit the skin's ability to produce Vitamin D [2].

Season: The changing seasons notably impact Vitamin D synthesis. Vitamin D production is most efficient during the summer when the sun is at its zenith in the sky, leading to more prolonged and direct sunlight exposure. In contrast, during the winter, the sun is lower in the sky, resulting in reduced UVB intensity and shorter exposure periods. Consequently, individuals in higher latitudes may struggle to maintain adequate Vitamin D levels during the winter due to limited UVB exposure [22].

Time of day: The time of day at which sun exposure occurs also influences Vitamin D synthesis. UVB radiation is most intense when the sun is at its highest point in the sky, typically between 10 a.m. and 3 p.m. Sun exposure during these peak hours is more likely to result in efficient Vitamin D synthesis compared to early morning or late afternoon exposure when the sun's angle is lower [16].

Skin type: Skin pigmentation is a significant determinant of Vitamin D synthesis. People with lighter skin have less melanin, the pigment responsible for skin colour, and therefore require less UVB exposure to produce Vitamin D. In contrast, individuals with darker skin have more melanin, which acts as a natural sunscreen, reducing the penetration of UVB radiation. As a result, those with darker skin may need more extended sun exposure to generate the same amount of Vitamin D as individuals with lighter skin [23].

Age: Age can influence the skin's ability to produce Vitamin D. Older individuals may experience a reduction in the skin's capacity for Vitamin D synthesis due to decreased levels of 7-dehydrocholesterol, the precursor molecule required for Vitamin D synthesis. This reduction in precursor availability can hinder the skin's ability to convert UVB radiation into Vitamin D [24] efficiently.

Clothing and sunscreen Use: Using clothing that covers most of the skin and applying sunscreen can block UVB radiation, inhibiting Vitamin D synthesis. While protecting the skin from harmful UV radiation is essential to prevent sunburn and skin cancer, individuals may need to balance sun protection practices with the need for Vitamin D production. Strategies such as exposing uncovered skin for short periods or considering Vitamin D supplements may be necessary when sun protection is paramount [25].

Recommended Sunlight Exposure for Vitamin D Production

The recommended sunlight exposure for achieving adequate Vitamin D production in the skin is influenced by several factors, including geographical location, season, and individual characteristics such as skin type. While there is no one-size-fits-all guideline, health authorities generally suggest the following recommendations as a starting point:

Duration of exposure: Health authorities recommend approximately 10 to 30 minutes of midday sun exposure at least two to three times a week. This exposure should ideally target areas of the body readily exposed to sunlight, such as the face, arms, legs, or back. It's important to note that the exact duration may vary based on individual factors and conditions [26].

Timing: The recommendation of midday sun exposure is significant because UVB radiation is most intense when the sun is at its highest point in the sky, typically between 10 a.m. and 3 p.m. During this time, the sun's angle allows for more direct and effective UVB exposure, making it more conducive to Vitamin D synthesis [27].

Exposure without sunscreen or protective clothing: To optimise Vitamin D synthesis, the skin must remain exposed without sunscreen or protective clothing during the recommended exposure time. Sunscreen blocks UVB radiation, which is necessary for Vitamin D production, so its use during this period should be avoided. However, it's crucial to balance obtaining sufficient Vitamin D and protecting the skin from harmful UV radiation that can lead to sunburn and skin cancer. Therefore, short periods of unprotected sun exposure are typically recommended [28].

Adjustment for individual factors: The recommended exposure duration may need to be adjusted based on individual factors, including skin type and geographical location. Individuals with lighter skin may require a shorter duration of sun exposure to produce adequate Vitamin D, while those with darker skin may need more extended exposure. Moreover, individuals living at higher latitudes, especially in winter, may find obtaining sufficient UVB exposure for Vitamin D synthesis challenging due to reduced UVB intensity [29].

Consideration of health conditions: Individuals with certain health conditions or medications that affect Vitamin D metabolism should consult healthcare professionals to determine the most appropriate sunlight exposure recommendations. Additionally, individuals at higher risk of skin cancer or those with a history of excessive sun exposure should prioritise alternative sources of Vitamin D, such as dietary supplements [30].

Vitamin D status in surgical intensive care unit patients

Prevalence of Vitamin D Deficiency in SICU Patients

High prevalence: Research consistently indicates a high prevalence of Vitamin D deficiency among SICU patients. This deficiency can vary in severity, ranging from mild insufficiency to severe deficiency. The prevalence is noteworthy and underscores the significance of addressing Vitamin D status in critical care. Several factors contribute to the elevated rates of deficiency in SICU patients [31].

Variability by location: The prevalence of Vitamin D deficiency in SICU patients can vary significantly based on geographic location. Regions with limited sunlight exposure, such as those at higher latitudes or regions experiencing extended winter months, tend to have higher deficiency rates. In these areas, reduced sunlight exposure and lower UVB radiation levels can hinder the skin's ability to produce Vitamin D, increasing the risk of deficiency among patients admitted to the SICU [32].

Patient demographics: The prevalence of Vitamin D deficiency in SICU patients may also be influenced by patient demographics. Several demographic factors can play a role in shaping the prevalence rates, including:

Age: Older individuals may be more prone to Vitamin D deficiency due to reduced skin's capacity to synthesise Vitamin D, decreased outdoor activity, and potential comorbidities that affect Vitamin D metabolism [33].

Gender: Some studies have suggested that gender may impact Vitamin D status, with differences observed between male and female SICU patients. However, the specific reasons behind these gender differences require further investigation [34].

Underlying medical conditions: Patients admitted to the SICU often have a range of underlying medical conditions, some of which may affect Vitamin D metabolism. For example, individuals with chronic kidney disease or malabsorption disorders may be at a higher risk of Vitamin D deficiency [35].

Factors Contributing to Vitamin D Deficiency in SICU (Limited Sunlight Exposure, Comorbidities, Medications)

Vitamin D deficiency is prevalent among SICU patients, and several interrelated factors contribute to this deficiency, particularly in individuals who are already vulnerable due to their critical illness. Understanding these contributing factors is crucial for both identifying at-risk patients and implementing appropriate interventions:

Limited sunlight exposure, extended indoor stay: SICU patients often spend significant periods indoors or in dimly lit environments, which can severely limit their exposure to natural sunlight. This restriction occurs due to the necessity of close monitoring, interventions, and treatments within the hospital setting [36].

Immobility and bed rest: Many SICU patients require extended bed rest and limited mobility due to the nature of their illness or post-surgical recovery. This immobility can further reduce opportunities for sun exposure, as they may not have the chance to go outdoors or engage in activities that promote sunlight exposure [37].

Comorbidities: Underlying Medical Conditions: SICU patients often have underlying medical conditions or chronic illnesses that can contribute to Vitamin D deficiency. Conditions that affect fat absorption, such as celiac disease and Crohn's disease, can reduce the absorption of dietary Vitamin D, potentially leading to deficiency [38].

Renal dysfunction: Patients with renal dysfunction, common in critical care settings, may experience impaired Vitamin D activation in the kidneys, further exacerbating Vitamin D deficiency [39].

Medications

Glucocorticoids: Certain medications commonly administered in the SICU, such as glucocorticoids (steroids), can affect Vitamin D metabolism. They may impair the conversion of Vitamin D to its active form or lead to increased excretion of calcium, which can negatively impact bone health [40].

Anticonvulsants: Some anticonvulsant medications, like phenytoin, can accelerate the metabolism of Vitamin D, reducing its availability for physiological functions [41].

Antirejection drugs: Certain drugs used in transplant recipients, such as corticosteroids and immunosuppressants, can interfere with Vitamin D function and metabolism [42].

Malnutrition

Reduced dietary intake: SICU patients may experience malnutrition or reduced dietary intake during their stay, particularly if they have difficulty eating due to their condition or need surgery. Inadequate dietary Vitamin D intake can contribute to deficiency significantly if the SICU stay is prolonged [43].

Obesity

Sequestration in fat tissue: Vitamin D is a fat-soluble vitamin that can become sequestered in fat tissue. Patients with obesity may have lower circulating levels of Vitamin D because it becomes stored in adipose (fat) tissue, reducing its bioavailability for physiological functions [44].

Potential consequences of vitamin D deficiency in critically ill patients

Immune Dysfunction

Impaired immune response: Vitamin D regulates the immune system. Deficiency in Vitamin D can impair the immune response, potentially leading to compromised defence mechanisms against infections. This deficiency may increase the risk of infections, including nosocomial infections acquired in the hospital setting, which can be particularly concerning in the SICU [20].

Muscle Weakness and Falls

Muscle weakness: Vitamin D is essential for maintaining muscle strength and function. Deficiency can contribute to muscle weakness, a significant concern in critically ill patients who may already experience muscle wasting due to their condition [45].

Increased risk of falls: Muscle weakness resulting from Vitamin D deficiency can increase the risk of falls in critically ill patients. This risk is especially pertinent in the SICU, where patient mobility is often compromised, and falls can lead to additional complications [46].

Prolonged hospital stay: Association with Longer Hospital Stays: Some studies have suggested that Vitamin D deficiency may be associated with extended hospital stays and increased healthcare costs. Prolonged hospitalisation can have various implications, including higher healthcare resource utilisation and potential complications related to extended bed rest [47].

Complications of Chronic Diseases

Exacerbation of chronic conditions: Vitamin D deficiency can exacerbate chronic conditions commonly seen in critically ill patients. For example, it can worsen osteoporosis by impairing calcium absorption and bone health. In addition, it may contribute to cardiovascular complications, including hypertension and atherosclerosis, and exacerbate respiratory diseases such as asthma and chronic obstructive pulmonary disease (COPD) [35].

Mortality

Complex relationship with mortality: The relationship between Vitamin D deficiency and mortality in SICU patients is complex and requires further investigation. While some studies have suggested an association between lower Vitamin D levels and increased mortality risk, causality is challenging to establish due to the multifaceted nature of critical illness and its various contributing factors. Additional research is needed to clarify the impact of Vitamin D deficiency on mortality in this patient population [48].

Clinical implications of vitamin D in SICU patients

Impact of Vitamin D Status on Patient Outcomes (Infection Rates, Mortality, Length of Stay)

Infection rates

Immune function impairment: Emerging evidence suggests that Vitamin D deficiency may increase the risk of infections in SICU patients. Vitamin D plays a crucial role in immune system regulation; deficiency can impair immune function. This impairment may increase susceptibility to infections, including nosocomial infections acquired during hospitalisation [20].

Increased risk: Patients in the SICU are already vulnerable to infections due to the nature of their critical illness and the invasive procedures often required. Vitamin D deficiency may further elevate this risk, potentially leading to more frequent and severe infections [49].

Mortality

Mixed results: Several studies have investigated the relationship between Vitamin D status and mortality in critically ill patients, including SICU patients. However, the results of these studies are mixed and complex, making it challenging to draw definitive conclusions [50].

Potential association: Some research suggests that Vitamin D deficiency may be associated with increased mortality rates in SICU patients. While the exact mechanisms underlying this association require further investigation, it underscores the potential significance of Vitamin D in patient survival [51].

Causality and confounding factors: Determining a causal relationship between Vitamin D deficiency and mortality is challenging due to the multifaceted nature of critical illness and various contributing factors. Additionally, confounding variables, such as the severity of illness, comorbidities, and treatment strategies, can influence outcomes [52].

Length of stay

Association with longer hospital stays: Vitamin D deficiency has been linked to more extended hospital stays, including in the SICU. Prolonged hospitalisation can have economic and resource implications, making it an essential consideration for healthcare providers and institutions [53].

Potential contributing factors: The reasons for more extended hospital stays in Vitamin D-deficient patients may be multifactorial. Factors such as increased infection risk, slower recovery from critical illness, and potential complications related to muscle weakness and falls can all contribute to extended hospitalisation [54].

Role of Vitamin D Supplementation in SICU Care

Vitamin D supplementation strategies

Individualized approach: Healthcare providers may consider Vitamin D supplementation for SICU patients with documented deficiencies. The choice of supplementation strategy can vary and should be individualised based on several factors, including the patient's baseline Vitamin D levels, severity of the deficiency, comorbidities, and response to treatment [49].

Types of Vitamin D: Vitamin D2 (ergocalciferol) and Vitamin D3 (cholecalciferol) are two common forms of Vitamin D used for supplementation. Vitamin D3 is often preferred, as it is more effective at raising and maintaining Vitamin D levels [55].

Dosing regimens: The dosing regimens for Vitamin D supplementation can also vary. Providers may prescribe high-dose loading regimens followed by maintenance or lower daily doses, depending on the patient's needs and the severity of the deficiency [56].

Monitoring and individualization

Baseline assessment: Healthcare providers typically assess the patient's baseline Vitamin D levels through blood tests before initiating Vitamin D supplementation. This assessment helps determine the extent of deficiency and guides supplementation decisions [57].

Individualized dosing: The appropriate dosage and duration of Vitamin D supplementation should be individualised based on the patient's circumstances. Factors such as the severity of deficiency, comorbidities (e.g., renal dysfunction), age, and response to treatment all play a role in determining the most suitable supplementation regimen [58].

Regular monitoring: Ongoing monitoring of Vitamin D levels is essential during and after supplementation. Regular blood tests can help healthcare providers assess the patient's response to treatment and adjust as needed to maintain optimal Vitamin D levels [59].

Potential benefits

Reduced infection rates: Some studies have suggested potential benefits of Vitamin D supplementation in SICU patients, including potentially reducing infection rates. Vitamin D plays a role in immune system regulation, and supplementation may help enhance immune function, potentially reducing the risk of infections, including nosocomial infections acquired in the hospital [60].

Improved immune function: Vitamin D is known to modulate the immune response. Supplementing deficient patients may improve immune function, which can be particularly important in critically ill patients with compromised immune systems [20].

Complex relationship: It's important to note that the relationship between Vitamin D supplementation and clinical outcomes in the SICU is complex, and more research is needed to establish clear guidelines and best practices. The effectiveness of supplementation may vary among individuals, and the impact on mortality and other outcomes requires further investigation [61].

Evidence from Clinical Trials and Observational Studies

Clinical trials: Clinical trials examining the impact of Vitamin D supplementation on SICU outcomes are limited but growing. These studies aim to determine the efficacy of supplementation in improving patient outcomes, reducing infection rates, and decreasing mortality. Results from ongoing trials will help inform clinical practice [62].

Observational studies: Numerous observational studies have explored the association between Vitamin D status and outcomes in critically ill patients. While observational data provide valuable insights, they cannot establish causality. Nevertheless, these studies suggest adequate Vitamin D levels may have clinical benefits in the SICU setting [63].

Challenges and limitations: It is essential to recognise the challenges and limitations of existing research. Variability in study design, patient populations, and Vitamin D assessment methods can make it difficult to draw definitive conclusions. Moreover, the optimal Vitamin D threshold for SICU patients and the most effective supplementation strategies remain areas of active investigation [64].

Mechanisms underlying vitamin D's effects on SICU patients

Immunomodulatory Effects of Vitamin D

Innate immunity

Antimicrobial peptides: Vitamin D plays a crucial role in the innate immune system by enhancing the production of antimicrobial peptides, including cathelicidins and defensins. These peptides have potent antimicrobial properties, enabling them to combat many pathogens, including bacteria, viruses, and fungi [65].

Defence against infections: Vitamin D contributes to the body's defence mechanisms against infections by promoting the production of these antimicrobial peptides. Vitamin D's role in enhancing innate immunity is particularly relevant in critically ill SICU patients who are often susceptible to infections due to various factors, including invasive procedures and compromised immune function [66].

Adaptive immunity

T cells and B cells: Vitamin D modulates the adaptive immune response. It can influence the differentiation and function of T cells (including T helper and cytotoxic T cells) and B cells, critical players in the adaptive immune system [19].

Vitamin D receptors: Importantly, Vitamin D receptors are present on the surface of these immune cells, allowing for direct regulation. Vitamin D can exert its effects by binding to these receptors and influencing immune cell behaviour [20].

Balanced immune response: A balanced immune response is essential in critically ill patients. Vitamin D's involvement in regulating adaptive immunity may help prevent excessive immune activation, which can contribute to inflammatory conditions seen in critically ill patients [20].

Anti-inflammatory effects

Mitigating excessive inflammation: Vitamin D exhibits anti-inflammatory properties, which can help mitigate excessive immune responses. Vitamin D's anti-inflammatory action may be particularly relevant in conditions where inflammation is dysregulated, such as cytokine storms and hyperinflammatory states [38].

Potential clinical benefit: The anti-inflammatory effects of Vitamin D have raised interest in its potential to reduce the risk of inflammatory complications in critically ill patients, potentially improving clinical outcomes [67].

Anti-inflammatory properties

Reduction of pro-inflammatory cytokines: Vitamin D has been shown to reduce the production of pro-inflammatory cytokines, such as interleukin-6 (IL-6) and tumour necrosis factor-alpha (TNF-α), often elevated in critically ill patients. This anti-inflammatory effect may improve outcomes by preventing or mitigating systemic inflammation [68].

Modulation of the NF-κB pathway: Vitamin D can inhibit the activation of nuclear factor-kappa B (NF-κB), a transcription factor that regulates many inflammatory genes. By modulating this pathway, Vitamin D can help regulate the inflammatory response [69].

Role in maintaining calcium and phosphorus homeostasis

Calcium absorption: One of the classical functions of Vitamin D is to enhance the absorption of calcium from the intestines. In critically ill patients, maintaining calcium homeostasis is essential for neuromuscular, coagulation, and cardiac function [8].

Phosphorus regulation: Vitamin D also plays a role in phosphorus metabolism, closely linked to calcium homeostasis. Ensuring an adequate phosphorus supply is crucial for normal cellular function [70].

Potential links between vitamin D and other physiological processes relevant to SICU care

Muscle function: Vitamin D is essential for muscle health, and deficiency can lead to muscle weakness and impaired mobility. In the SICU, maintaining muscle strength is critical for patient recovery and rehabilitation [71].

Respiratory function: Emerging research suggests Vitamin D may affect respiratory health and lung function. In SICU patients with respiratory conditions, optimising Vitamin D status could support lung function [72].

Cardiovascular health: Vitamin D has been associated with cardiovascular health, including its potential influence on blood pressure regulation and endothelial function. Cardiovascular complications are relevant in SICU care, and Vitamin D may play a role in mitigating some of these risks [73].

Mental health: Vitamin D receptors are present in the brain, and there is ongoing research into the potential links between Vitamin D status and mental health. In the SICU, addressing mental health needs is integral to patient care [74].

Challenges and limitations

Difficulties in Assessing Vitamin D Status Accurately

Multiple biomarkers: Vitamin D status is typically assessed using serum 25-hydroxyvitamin D [25(OH)D] levels, but there is no universal consensus on the optimal cutoffs for deficiency, insufficiency, and sufficiency. Different laboratories may use varying reference ranges, complicating the interpretation of results [75].

Seasonal variability: Vitamin D levels can vary seasonally, with lower values during the winter and higher values in the summer due to differences in sunlight exposure. A single measurement may not accurately capture a patient's Vitamin D status [76].

Assay variability: Variability in laboratory assays can affect the accuracy and reproducibility of Vitamin D measurements. Standardisation of assays is an ongoing challenge [77].

Variability in Patient Responses to Vitamin D Supplementation

Individual differences: Patients may respond differently to Vitamin D supplementation based on genetic factors, comorbidities, medication use, and dietary habits. Identifying each patient's optimal dosage and duration of supplementation can be challenging [13].

Risk of toxicity: While rare, excessive Vitamin D supplementation can lead to Vitamin D toxicity, adversely affecting health. Monitoring Vitamin D levels during supplementation is essential to avoid potential harm [78].

Ethical Considerations Regarding Sunlight Exposure in SICU

Balancing sun exposure and patient care: Encouraging sunlight exposure in healthcare raises ethical considerations. The need to balance Vitamin D synthesis with the protection of critically ill patients from sunlight-related risks, such as skin damage and skin cancer, requires careful consideration [79].

Informed consent: If sunlight exposure is used as a therapeutic intervention, obtaining informed consent from patients or their representatives becomes crucial. Patients should be informed about sunlight exposure's potential benefits and risks [80].

Gaps in Current Research and Areas for Future Investigation

Clinical trials: Despite emerging evidence, there is a need for well-designed, randomised controlled trials (RCTs) specifically focused on Vitamin D supplementation in SICU patients. RCTs can provide more substantial evidence regarding the impact of supplementation on infection rates, mortality, and other outcomes.

Optimal dosage and duration: Research should aim to determine the optimal dosage and duration of Vitamin D supplementation for SICU patients, considering individual variability and clinical context.

Mechanistic studies: Further mechanistic studies are needed to elucidate the precise mechanisms through which Vitamin D influences immune function, inflammation, and other relevant physiological processes in SICU patients.

Long-term outcomes: Investigating the long-term effects of Vitamin D status and supplementation on post-SICU recovery, quality of life, and functional outcomes is essential for a comprehensive understanding of its impact.

Safety profile: A better understanding of the safety profile of Vitamin D supplementation in critically ill patients is necessary, including potential interactions with medications and the risk of toxicity.

Cost-effectiveness: Assessing the cost-effectiveness of Vitamin D screening and supplementation in the SICU setting is vital to inform healthcare resource allocation.

## Conclusions

In conclusion, the intricate relationship between Vitamin D and patient outcomes in the Surgical Intensive Care Unit (SICU) presents a multifaceted landscape. Through our exploration of Vitamin D's immunomodulatory effects, anti-inflammatory properties, and role in maintaining calcium homeostasis, it becomes evident that Vitamin D is a critical factor influencing the recovery and overall health of SICU patients. Despite the challenges and complexities surrounding accurate assessment and supplementation, the implications for clinical practice are substantial. Routine screening, individualised dosing strategies, and patient education can help optimise care in the SICU. Furthermore, the potential for personalised Vitamin D supplementation strategies aligns with the evolving paradigm of tailored medical interventions. Ultimately, the Sunlight-Vitamin D Connection holds significant promise for improving patient outcomes in the SICU, underscoring the importance of ongoing research and the potential to enhance the well-being of critically ill individuals in this critical care setting.
